# Supplementary material for: Intermediate progenitors support migration of neural stem cells into dentate gyrus outer neurogenic niches
Source: eLife. 2020 Apr 3;9:e53777. doi: 10.7554/eLife.53777 (PMC7159924; doi:10.7554/eLife.53777)
Supplement: Source data 1. [file elife-53777-data1.docx]

| **Source Data 1. Live-cell multiphoton 4D datasets of embryonic, postnatal, and adult IPs and NSCs in the Dentate Gyrus.** | | | | | | | |
| --- | --- | --- | --- | --- | --- | --- | --- |
| Age | **Animal** | **Slices^1^** | **Hours** | **Mitoses^2^** | | **Cell Tracks** | **Dynamics** |
|  |  |  |  | INPs | NSCs |  |  |
| E16.5 | *tgTbr2GFP* | 3 | 24h:30m | 36 | 11 | 142 | M, F, T |
| E17.5^3^ | *kiTbr2GFP* | 2 | 11h:18m | 46 |  | 63 | M, F, T |
| E17.5 | *NesCreERT2:Ai14/kiTbr2GFP* | 2 | 13h:35m | 5 | 4 | 14 (NSCs) 31 (IPs) | M, F, T |
| E17.5*^ef^* | *NesCreERT2:Ai14/kiTbr2GFP* | 2 | 4h:48m |  |  | 99 (IPs) | M, F |
| E18.5 | *kiTbr2GFP* | 2 | 14h:44m | 29 | 2 | 238 | M, F |
| P3-4 | *tgTbr2GFP* | 3 | 8h:0m | 6 |  | 128 | M, F |
| P4-5*^ef^* | *tgTbr2GFP* | 2 | 2h:26m | 5 |  | 21 | M, F |
| P7^4^ | *tgTbr2GFP* | 1 | 2h:33m | 1 |  |  | M, F |
| P12 | *tgTbr2GFP* | 2 | 4h:11m | 2 |  | 15 | m, F |
| P14 | *tgTbr2GFP* | 2 | 7h:52m | 3 |  | 1 | m, F |
| P21 | *tgTbr2GFP* | 4 | 11h:14m | 2 |  | 3 | m, F |
| P22 | *tgTbr2GFP* | 2 | 7h:24m | 2 |  |  | m, F |
| P22 | *kiTbr2GFP* | 1 | 2h:30m |  |  |  | F |
| P35 | *NesCreERT2:Ai14* | 2 | 8h:23m |  | 2 |  |  |
|  | ***total*** | ***28*** | ***~123h:30m*** | ***137*** | ***17*** | ***755*** |  |
|  |  |  |  | ***= 154 total live mitoses*** | |  |  |
| *Abbreviations: M, migrations (m, limited migrations); F, filopodial-like process extensions, retractions, branching; T, torsional morphogenics;* | | | | | | | |
| *E, embryonic day; P, postnatal day; tg, transgenic; ki, knockin; h, hours; m, minutes* | | | | | | | |
| *^1^successfully imaged slices only; ^2^Mitoses refers to IPs (GFP+) and NSCs (GFP- in tgTbr2GFP/kiTbr2GFP models and/or tdTom+ in NesCreERT2:Ai14 model); ^3^slices prepared at E16.5 and cultured 1 day in vitro to E17.5; ^4^from Hodge et al., 2012; ^ef^ en face* | | | | | | | |
